# A Suggestion

**Published:** 1864-07

**Authors:** 


### A SUGGESTION.

*Dr. J. F. Miner, Editor Buffalo Medical and Surgical Journal:*

Dear Sir:—The recent report of Drs. Krombein and Dingler, upon the Trichina Spiralis disease in Erie County, as also the able paper upon the same subject by W. Keller of Darmstadt, re-published in the *American Journal of Medical Sciences*, suggests to my mind a solution of the vexed question of the National Hotel disease at Washington, which several years ago proved so fatal, and which defied all attempts at analysis and treatment. If any of your readers are sufficiently familiar with that disorder to judge of the comparative symptoms between it and those produced by trichina, their opinion is solicited as to the correctness of the surmise of

Yours Respectfully,

THOS. F. ROCHESTER.

MORRIS, June 26th, 1864.

Editor of Buffalo Medical and Surgical Journal:

DEAR SIR:—Inclosed find a case that occurred about twelve miles distant from me, which, if you find worthy, you will note in your Journal, and please give us through its columns your opinion, &c.

J. W. STILLE.

James H. Birdsall, a young man, previously in good health, was attacked with inflammation of the left lung about the first of April last. For several days his life was considered in imminent danger. At length the violence of the symptoms was overcome and the hope of recovery indulged. About this time improper exposure kindled into activity the smothered embers of disease. Frequent chills, followed by fever and attended with severe pain, and laborious breathing, which became more and more aggravated during a period of three weeks, led us to suspect the formation of abscess. On the sixteenth of May a consultation was held, the presence of fluid to a large extent positively detected, and an operation for its removal recommended. The day following an opening was made between the ribs of the left side and nearly three quarts of pus escaped, with decided and immediate relief to the patient. The wound was then closed, with directions that it be re-opened as often as symptoms indicated the accumulation of fluid in the cavity of the abscess. It became necessary to remove the dressings daily in order to give vent to the accumulation for fourteen days, the discharge averaging about a pint a day, when nature, exhausted, finally gave way.

A *post mortem* examination disclosed a cavity of large dimensions between the lung and the walls of the chest, the lung itself, together with the heart, having been forced far toward the opposite side by the continued pressure of the accumulated fluid. The left lung, instead of being destroyed by suppuration, as was supposed, had become consolidated by the inflammatory process; all appearance of air cells or bronchial tubes having been entirely obliterated. The pericardium was adherent to the heart throughout and both lung and heart so firmly attached to the mediastinum as to present the semblance of one solid mass. The right lung was compressed by the displacement of the mass to one-half its natural thickness.*

---

* We should presume that the above case was one of pleuritic inflammation, ending in *empyema*, which is not an infrequent termination. Whether the fluid is serum or pus, the physical signs are the same. Abscess of the lung is said to sometimes form, and by rupture, produce some such results as above described. The condition of the lung, in this case, was probably produced mostly by pressure.—ED.

